# Electrochemical Performance of MnO_2_/Graphene Flower-like Microspheres Prepared by Thermally-Exfoliated Graphite

**DOI:** 10.3389/fchem.2022.870541

**Published:** 2022-04-08

**Authors:** Xuyue Liu, Bing Liang, Xiaodong Hong, Jiapeng Long

**Affiliations:** ^1^ School of Material Science and Technology, Shenyang University of Chemical Technology, Shenyang, China; ^2^ School of Materials Science and Energy Engineering, Foshan University, Foshan, China

**Keywords:** MnO_2_, thermally-exfoliated graphite, supercapacitors, electrochemical performance, flower-like microspheres

## Abstract

To enhance the electrochemical performance of MnO_2_/graphene composite, herein, thermally-exfoliated graphite (TE-G) is adopted as a raw material, and a hydrothermal reaction is conducted to achieve the exfoliation of TE-G and the loading of MnO_2_ nanosheets. Through optimizing the TE-G/KMnO_4_ ratio in the redox reaction between carbon and KMnO_4_, flower-like MnO_2_/G microspheres (MnO_2_/G-10) are obtained with 83.2% MnO_2_ and 16.8% residual graphene. Meanwhile, corresponding MnO_2_/rGO composites are prepared by using rGO as raw materials. Serving as a working electrode in a three-electrode system, MnO_2_/G-10 composite displays a specific capacitance of 500 F g^−1^ at 1 A g^−1^, outstanding rate performance, and capacitance retention of 85.3% for 5,000 cycles. The performance is much better than that of optimized MnO_2_/rGO composite. We ascribe this to the high carbon fraction in TE-G resulting in a high fraction of MnO_2_ in composite, and the oxygen-containing groups in rGO reduce the resulting MnO_2_ fraction in the composite. The superior electrochemical performance of MnO_2_/G-10 is dependent on the hierarchical porous structure constructed by MnO_2_ nanosheet arrays and the residual graphene layer in the composite. In addition, a supercapacitor assembled by TE-G negative electrode and MnO_2_/G positive electrode also exhibits superior performance. In consideration of the low cost of raw materials, the MnO_2_/G composite exhibits great application potential in the field of supercapacitors.

## Introduction

Among the existing energy storage devices, the supercapacitor is an important device for high power density, rapid charge/discharge, and long cycling life. The fabrication of electrode materials is a major task for developing high-performance supercapacitors (Raj et al., 2020; Oncu et al., 2021; Zhang et al., 2021). To achieve the rapid transport and transfer of ions/electrons, various carbon materials have been developed in the field of supercapacitors, including carbon nanotubes (Lei et al., 2020), graphene (Sha et al., 2021), carbon nanosheets (Sevilla and Fuertes, 2014), porous carbon (Zhao et al., 2020), carbon fibers (Srimuk et al., 2015), and so on. Nevertheless, the poor specific capacitance of these carbon materials affects their wide application in supercapacitors, due to the electrical double layer capacitance (EDLC) feature (Sevilla and Fuertes, 2014; Ferrero et al., 2015). To enhance the specific capacitance, carbon materials have been hybridized with various metal oxides for introducing high pseudocapacitance (Yan et al., 2014, 2021). Among those transition metal oxides, MnO_2_ has been regarded as the most promising electrode material, due to the large theoretical specific capacitance of 1370 F g^−1^, natural abundance, and low price (Xu et al., 2007, 2018; Zhang et al., 2020c).

Lots of methods have been reported to prepare MnO_2_/rGO composites, such as the chemical precipitation method (Gong et al., 2021), alcohol infiltrated substrate method (Zhang et al., 2020b), and hydrothermal route (Liu et al., 2015). Among these methods, the hydrothermal method is the most convenient way for synthesizing MnO_2_/rGO composites. During a hydrothermal process, a redox reaction takes place between carbon and KMnO_4_, and MnO_2_ nanostructures are uniformly generated on graphene nanosheets, with the consumption of a certain amount of carbon (Ping et al., 2019, 2; Hong et al., 2021; Wang T. et al., 2021). In this respect, by using sulfur-reduced graphene oxide (RGO-S) as raw materials, Tarimo et al. (Tarimo et al., 2020) synthesized RGO-S/MnO_2_ composite via a hydrothermal method, and the optimized RGO-S/MnO_2_ composites had a low capacitance (180.4 F g^−1^). Yang et al. (Yang et al., 2012) prepared rGO firstly by using graphene oxide (GO) and then synthesized urchin-like MnO_2_ on rGO nanosheet through a hydrothermal reaction under the presence of KMnO_4_. The optimized rGO/MnO_2_ composites exhibited a high capacitance of 263 F g^−1^. Moreover, Liu et al. (Liu et al., 2014) prepared GO firstly by Hummers method and then synthesized MnO_2_-GO composites via hydrothermal reaction. The MnO_2_-GO composite presented a capacitance of 213 F g^−1^ at 0.1 A g^−1^. From these works about MnO_2_/graphene composites, the graphene in composites is usually derived from GO prepared by Hummers method (Vimuna et al., 2020). In addition, the resulting MnO_2_/rGO composites deliver the specific capacitance of less than 300 F g^−1^, which further limits the development of high-performance supercapacitors. Up to now, there is no report about MnO_2_/graphene composites prepared by using expandable graphite as raw materials.

In view of the larger specific surface area, lower oxygen content, more complete lamellar structure, and low cost and easy preparation of thermally-exfoliated graphite (TE-G), herein, TE-G was adopted as raw materials, and a hydrothermal reaction was performed to fabricate MnO_2_/graphene composite through a redox reaction between KMnO_4_ and C. Most importantly, the hydrothermal reaction achieves the exfoliation of TE-G. As a result, flower-like MnO_2_/graphene microspheres were produced, in which, the residual graphene layer was wrapped by abundant thin MnO_2_ nanosheets. The optimized MnO_2_/graphene microspheres exhibited excellent electrochemical performance in supercapacitors. To verify the performance advantage of TE-G in preparing MnO_2_/graphene composite, various MnO_2_/rGO composites were fabricated by using GO as reactants, and corresponding electrochemical performance was investigated. Compared with rGO, the MnO_2_/G composite prepared with TE-G as raw material shows better performance and a more convenient method.

## Experiment

### Materials

Potassium chloride (KCl), Expandable graphite (EG, 80 mesh), and potassium permanganate (KMnO_4_) were obtained from Tianjin Damao Chemical Reagent Factory.

### Preparation of Thermally-Exfoliated Graphene

Thermally-exfoliated graphite (TE-G) was synthesized according to our previous work (Liu et al., 2021). Specifically, EG was heated at 500°C for 100 min under N_2_ to obtain thermally-exfoliated graphene (TE-G).

### Preparation of MnO_2_/Graphene (MnO_2_/G) Composites

In a typical synthesis, 1.0 g KMnO_4_ was put into deionized water (80 ml) and stirred for 30 min to produce a uniform solution. Meanwhile, different amounts of TE-G powders were put into the KMnO_4_ solution and stirred for 30 min, and then, the mixture was put into a stainless-steel autoclave. The hydrothermal reaction was conducted at 180°C for 15 h. The production was filtered, rinsed repeatedly by deionized water, and dried at 60°C for 12 h to obtain MnO_2_/G composites. The redox reaction equation of C and KMnO_4_ can be described as: 4MnO_4_^−^ + 3C + H_2_O → 4MnO_2_ + CO_3_^2−^ + 2HCO_3_^−^. According to the equation, the theoretical mass ratio of KMnO4 and C can be calculated as 1/17.7. Therefore, to change the MnO_2_ fraction in the MnO_2_/G composite, the KMnO_4_/TE-G mass ratio was set as 5, 10, and 20, and the resulting composites were coded as MnO_2_/G-5, MnO_2_/G-10, and MnO_2_/G-20. In addition, the hydrothermal reaction of TE-G in deionized water and in KCl solution was carried out under the same condition, and the resulting samples were coded as TE-G-H_2_O and TE-G-KCl, respectively. The rGO was used to prepare MnO_2/_rGO composites. The ratio of KMnO_4_/rGO was kept the same as the ratio of KMnO_4_/TE-G composites, and the sample was named MnO_2_/rGO-5, MnO_2_/rGO-10, and MnO_2_/rGO-20.

### Testing and Characterization

The field-emission scanning electron microscopy (FE-SEM; SU8010) and transmission electron microscopy (TEM; JEM-2100) were used to observe the morphologies of samples. The crystallographic feature was performed by X-ray diffraction (XRD; D8-Advance) with Cu Kα radiation source. X-ray photoelectron spectra (XPS) were recorded by using a Thermo Scientific K-Alpha XPS spectrometer. The working voltage was 12 kV, and the X-Ray source was Al Kα. Pore size distribution and the specific surface area were tested by using the SSA-7000 device, according to the BJH model and BET method.

### Electrochemical Performance

A three-electrode system was used to test the electrochemical performance of samples in an electrolyte of 6 M KOH. The poly (vinylidene fluoride)/acetylene black/active materials were weighed at the ratio of 5:10:85, and dissolved in N-methyl-2-pyrrolidone (NMP) to prepare a slurry. Foam nickel (1 × 1 cm^2^) was used to support the slurry and served as the working electrode. The platinum sheet was acted as the counter electrode, and the saturated calomel electrode (SCE) was used as the reference electrode. A CHI 660E electrochemical workstation (Shanghai Chenhua Co. Ltd.) was used to test electrochemical impedance spectroscopy (EIS), galvanostatic charge/discharge curves (GCD), cycling stability, and cyclic voltammetry (CV) curves. Asymmetric supercapacitor (ASCs) devices were assembled by using TE-G as the negative electrode and MnO_2_/G composite as a positive electrode with 6 M KOH electrolyte. The separator was glass fiber filter paper. In the ASCs device, Formula I (Wang et al., 2018): R = *m* ^+^/m^−^=(C^−^×ΔV^−^/(C^+^×ΔV^+^) can be applied to obtain the ratio of positive/negative electrode material. Formula II (Brousse et al., 2007; Hong et al., 2021): C=I×Δt/ΔV, was employed in calculating the specific capacitance (C) in a three-electrode system. Formula III (Brousse et al., 2007): Cs = 4C/M, Formula IV (Li et al., 2021): E = 0.5C (ΔV)^2^/3.6 and Formula V (Brousse et al., 2007): P = E/Δt can be applied to obtain specific capacitance (Cs), the energy density (E) and power density (P) of the ASCs, respectively.

## Results and Discussion

### Preparation Process of MnO_2_/G Composite

**FIGURE 1:**
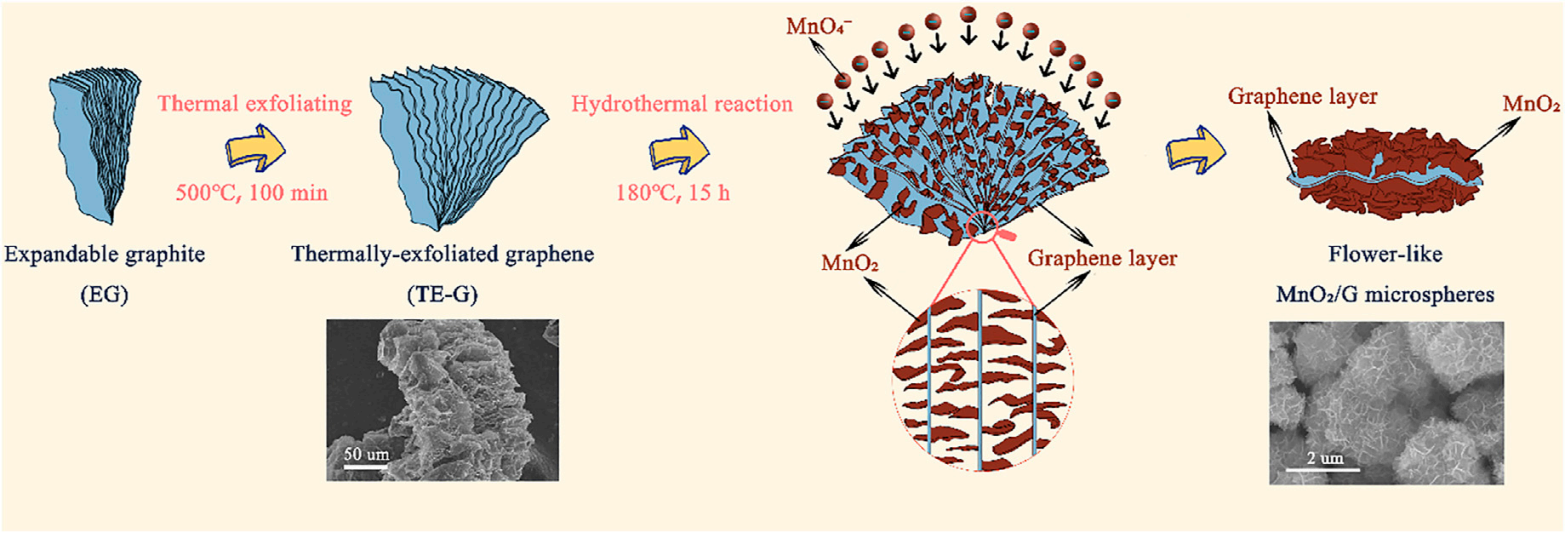
Schematic presentation of the preparation of accordion structure TE-G and flower-like MnO_2_/G microspheres, and the inset SEM images presenting the morphology of TE-G and MnO_2_/G composites respectively.

Figure 1 exhibits the preparation process of flower-like MnO_2_/G microspheres. Firstly, under the presence of N_2_, the expandable graphite (EG) was heated at 500°C to prepare TE-G. Under a high temperature, the intercalation agent in EG expands and violently decomposes, resulting in a large amount of gas spilling and forming micropores, mesopores, and macropores. As shown in Figure 1, TE-G shows an accordion structure with a thick lamella. Moreover, abundant cavity structures can be observed on TE-G. Secondly, under a hydrothermal process, KMnO_4_ reacts with C to generate MnO_2_, in which, each single-layer of graphene in TE-G reacts with KMnO_4_ and is then wrapped by abundant MnO_2_ nanosheets. The loading of MnO_2_ thick nanosheets on graphene layers leads to the exfoliation of TE-G. From the inset SEM image, after the redox reaction, graphene nanosheets were wrapped by MnO_2_ nanosheet arrays in different directions to produce flower-like microspheres. Compared with rGO, the consumption of graphene nanosheets and the generation of thick MnO_2_ layers lead to the delamination of TE-G. In order to confirm the advantage of MnO_2_/G composite, corresponding MnO_2_/rGO composites were prepared, and the microstructure and electrochemical performance were investigated.

### Microstructure of MnO_2_/G Composites

**FIGURE 2:**
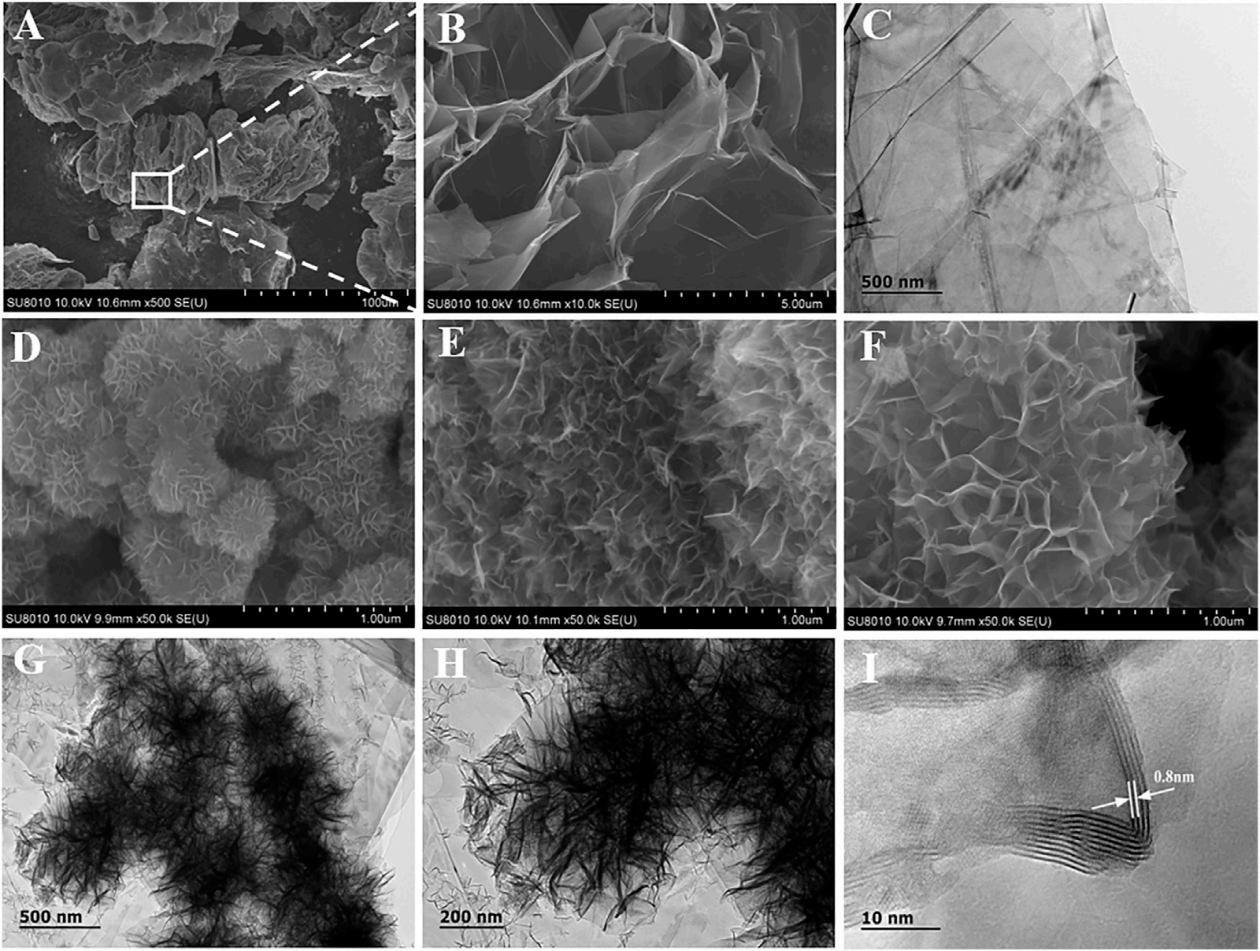
The microstructure of pure TE-G **(A–C)** and the composites with the KMnO_4_/TE-G ratio of 5 **(D)**, 20 **(E)**, and 10 **(F–I)** by SEM and TEM.

The morphologies of TE-G and different MnO_2_/G composites samples were characterized by using TEM and SEM. From Figure 2A,B, pure TE-G presents an accordion structure with a large number of holes, and Figure 2C indicates the stacking structure of abundant graphene nanosheets. From these MnO_2_/G samples, under a low ratio of KMnO_4_/TE-G, a few graphene sheets in TE-G participate in the redox reaction with KMnO_4_. Hence, a few MnO_2_ nanosheets are generated on the graphene surface (Figure 2D). When the ratio of KMnO_4_/TE-G increases to 10, dense MnO_2_ nanosheet arrays are generated in all directions of graphene nanosheets, presenting a flower spherical structure (Figure 2F). From the high magnification SEM in Figure 2F, the resulting MnO_2_ nanosheets arrays exhibit a honeycomb-like structure. The results show that the redox reaction between carbon and KMnO_4_ produces MnO_2_ nanosheets on graphene, which achieves the delamination of TE-G. From Figure 2G,H, there are dense MnO_2_ nanosheets arrays dispersed on the graphene surface. Moreover, the TEM image also shows the connection of different MnO_2_/G flower spheres, which may be resulted from the fracture of large graphene nanosheets during the high-temperature hydrothermal reaction process. As shown in Figure 2I, we can observe the diffraction fringes of MnO_2_ on the graphene surface. The fringe spacing of ∼0.8 nm corresponds to the (001) facet of δ-MnO_2_ (Wang J. et al., 2021). When the ratio of KMnO_4_/TE-G reaches 20, excessive MnO_2_ nanosheets are generated and piled up on the surface of the MnO_2_/G composite (Figure 2E).

**FIGURE 3:**
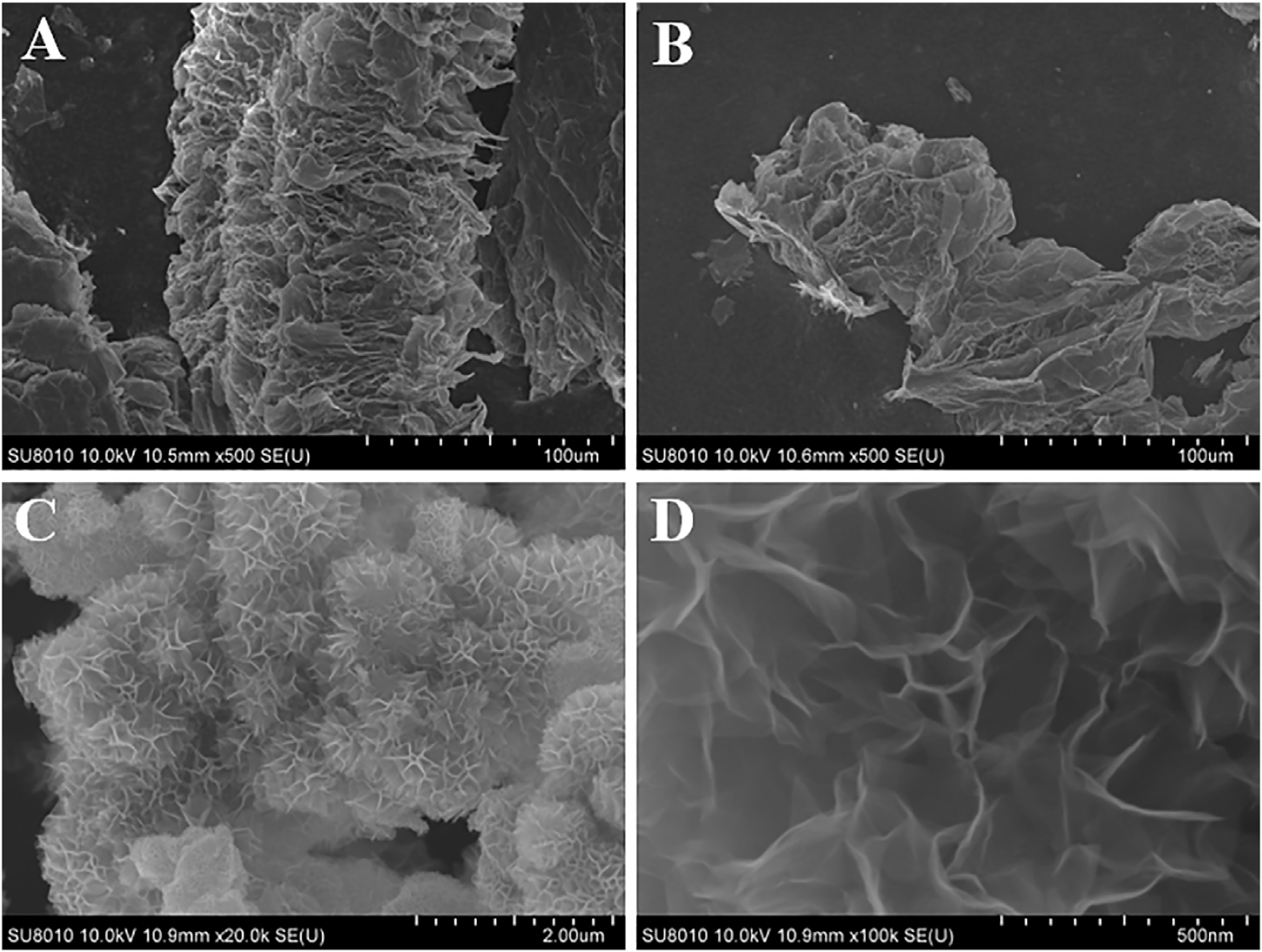
The microstructure of TE-G-H_2_O **(A)**, TE-G-KCl **(B)**, and MnO_2_/rGO composites **(C,D)**.

In order to prove that the exfoliating of TE-G is related to the KMnO_4_-assisted hydrothermal reaction, two controls are designed by using only deionized water and KCl solution, respectively. In the absence of KMnO_4_, the hydrothermal reaction cannot exfoliate the TE-G. As shown in Figure 3A, the accordion structure is kept the same as pure TE-G (Figure 2A). Under the presence of K^+^ derived from KCl, the resulting TE-G also keeps the same structure with pure TE-G in Figure 2A. Therefore, the exfoliation of TE-G is dependent on KMnO_4_-assisted hydrothermal reaction, and the *in-situ* reaction between graphene nanosheet and KMnO_4_ consumes carbon and introduces MnO_2_ nanosheet arrays, which effectively exfoliate TE-G. To disclose the advantage of TE-G in preparing MnO_2_/G composites, rGO was used as reductants, and resulting MnO_2_/rGO composites were shown in Figure 3C,D. The MnO_2_/rGO composite shows the same flower spheres as MnO_2_/G composite (Figure 2D–F). The result indicates that the reaction between rGO and KMnO_4_ is kept the same as the reaction between TE-G and KMnO_4_, that is, the redox reaction of graphene nanosheets and KMnO_4_. However, the major difference between the two reactions is the carbon precursors. TE-G has condensed graphene nanosheets with no oxygen-containing groups, while rGO is the exfoliated graphene containing oxygen-containing groups. Compared with rGO, TE-G has a low cost and high carbon content, which would consume more KMnO_4_ and introduce much more MnO_2_, while some rGO nanosheets are not wrapped by MnO_2_ nanosheets arrays (Figure 3C), and much more MnO_2_ nanosheets would enhance the electrochemical performance of MnO_2_/G composites, which will be discussed further.

### Elemental Distribution of MnO_2_/G Composites

**FIGURE 4:**
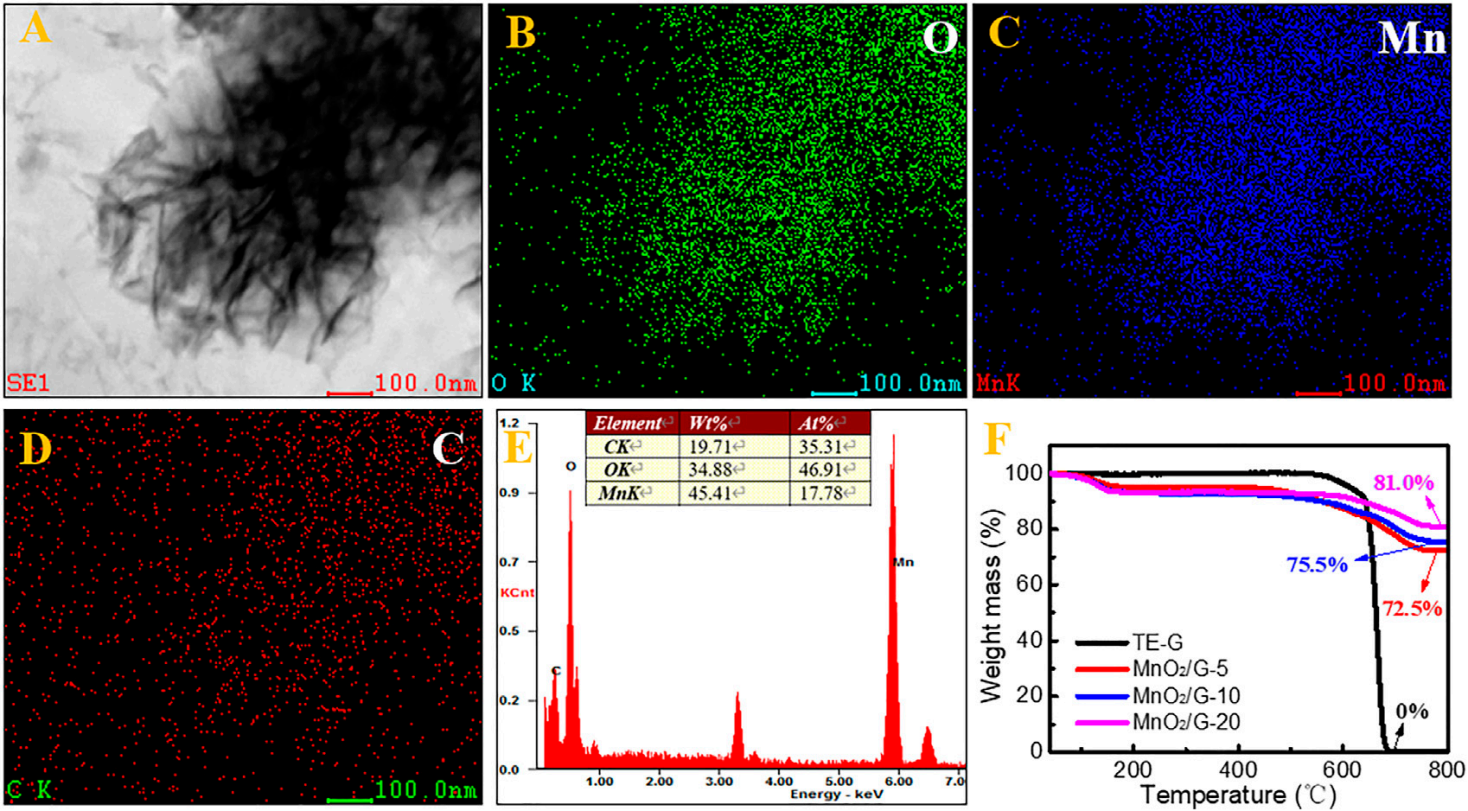
The microstructure of the MnO_2_/G-10 composite observed by STEM **(A)**, the elemental mapping of O **(B)**, Mn **(C)**, and C **(D)**, corresponding elemental fraction **(E)**, and thermogravimetric curves of TE-G and MnO_2_/G composites **(F)**.

Elemental mapping and EDS were conducted to verify residual graphene in MnO_2_/G composite. MnO_2_ nanosheets (Figure 4A) keep the same with the SEM morphology (Figure 2F). The distribution of O is in accordance with the Mn (Figure 4C), which reflects the generation of MnO_2_. In addition, the dispersed C signals verify the residual graphene in the MnO_2_/G composite. From Figure 4E, the C content is at 35.31%, further demonstrating the residual carbon derived from graphene. To detect the precise carbon content in composite, TG curves of TE-G, MnO_2_/G-5, MnO_2_/G-10, and MnO_2_/G-20 are given in Figure 4F. When the temperature is higher than 600°C, TE-G begins to decompose, and no residual carbon remains at 800°C. Compared with TE-G, the residual fractions of three composites are 72.5, 75.5, and 81.0% at 800°C in air. Based on the principle in Ref. (Wang J. et al., 2021), the final product of MnO_2_/G composite is Mn_2_O_3_ at 800°C. According to the same Mn content, we can calculate the fraction of MnO_2_, that is, 80.0, 83.2, and 89.3%, respectively. The residual carbon fractions in MnO_2_/G-5, MnO_2_/G-10, and MnO_2_/G-20 are 20.0, 16.8, and 10.7%, respectively. Therefore, the TG result affirms the incomplete reaction of carbon (TE-G), and residual graphene nanosheet still exists in the MnO_2_/G composite.

Fourier transform infra-red (FTIR) was provided in Supplementary Figure S1, the peak at ∼3,425 cm^−1^ is attributed to the O-H vibration of GO or rGO. The peaks of ∼1,633 cm^−1^ and 1,313 cm^−1^ correspond to the stretching and bending vibration of C-O, respectively. Compared with GO or rGO, there are a few oxygen-containing groups in TE-G, which is conducive to the redox reaction between TE-G and KMnO_4_, and a high fraction of carbon would consume a large amount of KMnO_4_ and generate many more MnO_2_ nanosheets. To verify the high fraction of MnO_2_ in MnO_2_/G composites, the TG curves of MnO_2_/rGO-5 and MnO_2_/rGO-20 were measured to obtain the content of MnO_2_ in MnO_2_/rGO composites. As given in Supplementary Figure S2, the residual fractions of MnO_2_/rGO-5 and MnO_2_/rGO-20 are 57.7 and 67.0%, respectively. Therefore, the fraction of MnO_2_ can be calculated as 63.6 and 73.8%, respectively. The result shows that the MnO_2_ content of MnO_2_/rGO is much lower than that of the corresponding MnO_2_/G composite. The reason can be ascribed to the low C fraction in rGO, resulting in fewer MnO_2_ nanosheets.

### Crystal Structure and Surface Chemistry of TE-G and MnO_2_/G

**FIGURE 5:**
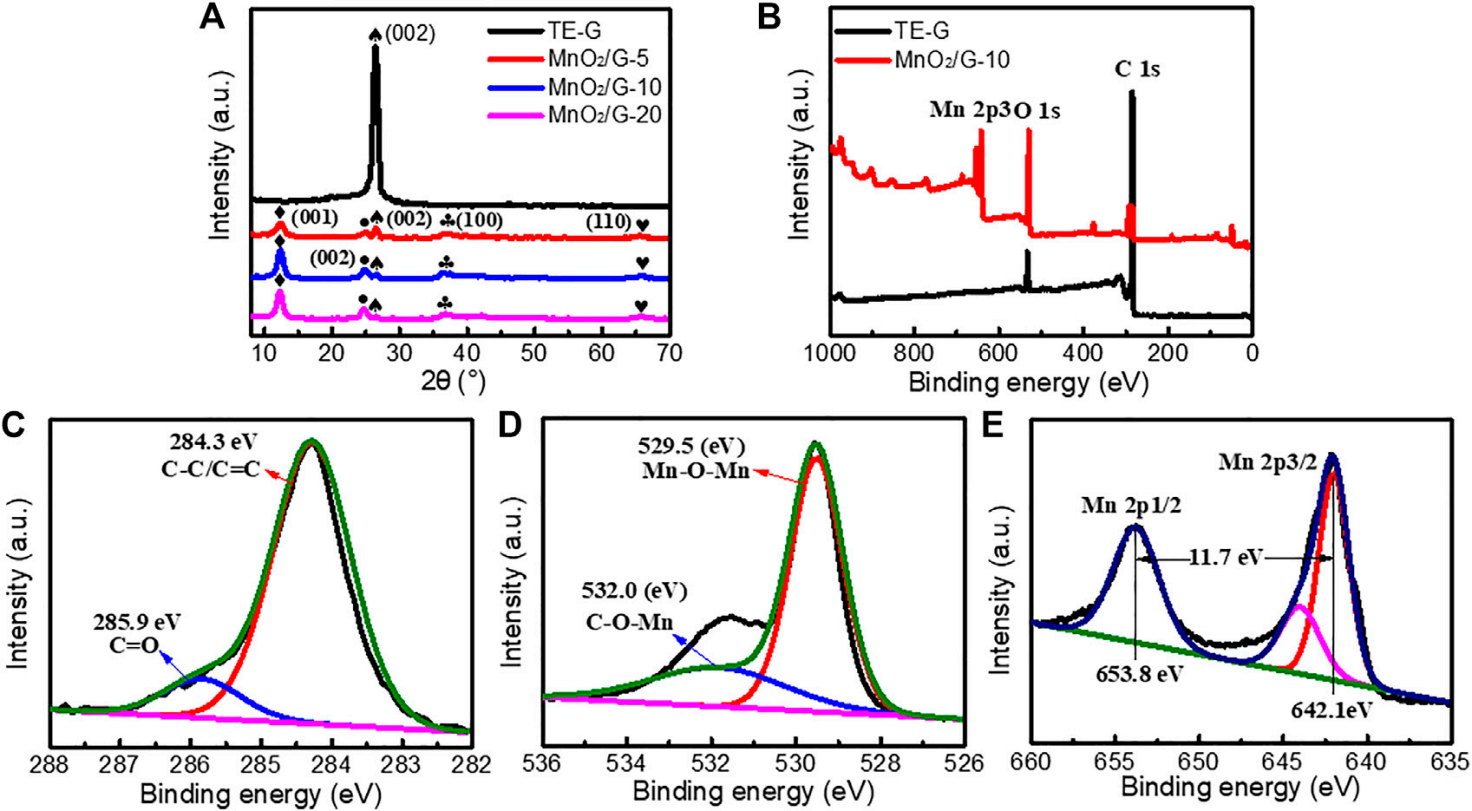
The XRD patterns **(A)** and XPS survey spectra **(B)** of pure TE-G and MnO_2_/G-10 sample, and the magnified C **(C)**, O **(D)**, and Mn **(E)** XPS spectrum.

In order to analyze the crystal structure of samples, XRD testing was performed. As given in Figure 5A sharp diffraction peak at 26.4° is attributed to the (002) crystal plane of TE-G (Thommes and Cychosz, 2014). According to the Bragg equation: 2dsinθ = nλ, the layer spacing d is calculated as 0.34 nm. After reacted with KMnO_4_, four peaks can be observed at 12.2°, 24.7°, 36.6°, and 65.6°, these peaks correspond to the (001), (002), (100), and (110) facets of δ-MnO_2_ (JCPDS # 80–1098) (Wei et al., 2012; Zhu et al., 2017). When the amount of KMnO_4_ increased, the (002) peak of carbon (TE-G) at 26.4° disappears, which is assigned to the loading of MnO_2_ thick nanosheets on graphene layers leads to the exfoliation of TE-G. This phenomenon indicates the consumption of TE-G and results in a low fraction of carbon in MnO_2_/G composites. Compared with MnO_2_/G composite, hydrothermally-treated TE-G samples under deionized water or KCl both show a sharp diffraction peak at 26.4° (as shown in Supplementary Figure S3), which confirms that the TE-G cannot be exfoliated by H_2_O or KCl under hydrothermal reaction. Therefore, the exfoliation of TE-G is dependent on KMnO_4_. In addition, the chemical bonds and valance state of TE-G and MnO_2_/G-10 samples were characterized by XPS. From the general spectra in Figure 5B. The TE-G spectrum shows the peaks of C and O elements. After reacting with KMnO_4_, the peak of C weakens, and the peaks of O and Mn elements are stronger obviously, due to the generation of MnO_2_ and the consumption of TE-G nanosheets. Figure 5C–E shows the magnified C, O, and Mn spectrum. From Figure 5C, the magnified C 1s spectrum can be divided into two peaks at 284.3 and 285.9 eV, which are attributed to the C-C/C=C bond and C=O bond (Yang et al., 2020), respectively. The high-resolution O 1s can be convoluted into two main peaks at 532.0 and 529.5 eV (Figure 5D) corresponding to the bond of C-O-Mn generated between graphene and MnO_2_, and the bond of Mn-O-Mn in MnO_2_ (Yang and Park, 2018; Yang et al., 2020). From the magnified Mn 2p spectrum (Figure 5E), the 2p orbital of Mn has two major peaks at 642.1 and 653.8 eV, corresponding to Mn 2p_3/2_ and Mn 2p_1/2_. The distance of the two peaks is around 11.7 eV, which reflects the +4 valence of the Mn element (Yang and Park, 2018; Li et al., 2020). Hence, the MnO_2_ in the composite is further proved by XPS.

### BET Analysis

**FIGURE 6:**
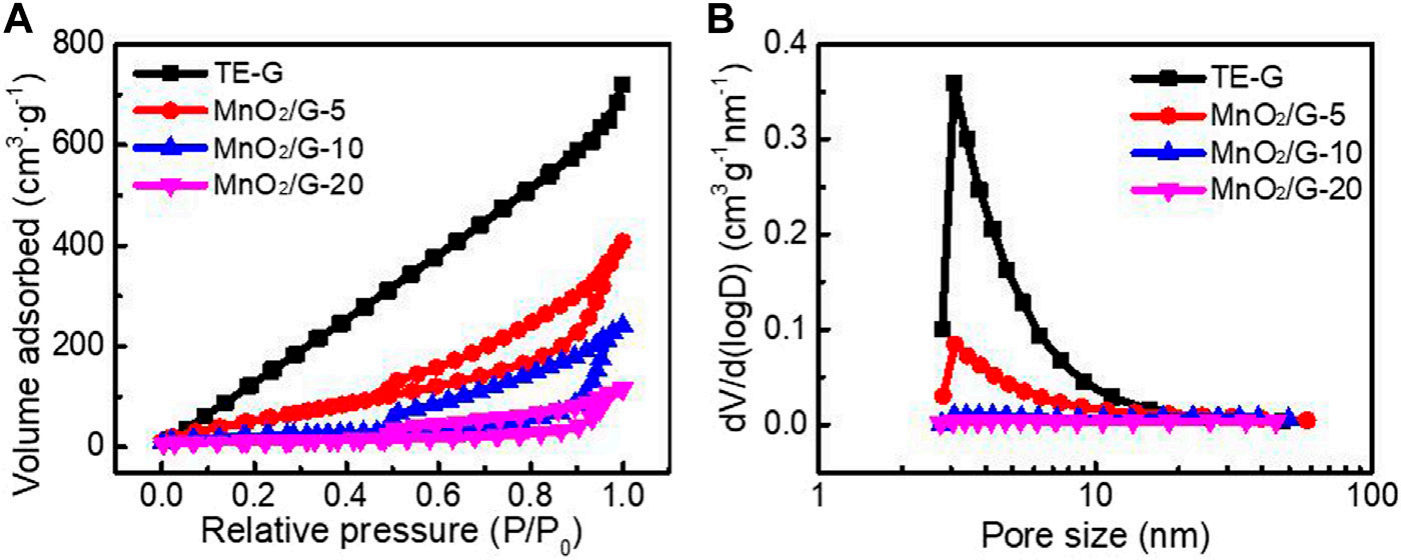
N_2_ adsorption-desorption isotherms **(A)** and pore size distribution **(B)** of pure TE-G, MnO_2_/G-5, MnO_2_/G-10, and MnO_2_/G-10 composite.

Figure 6 shows the N_2_ adsorption-desorption isotherms curves and pore distribution of different samples according to the BJH model and BET method. TE-G exhibits a high adsorption capacity and a big specific surface area at low pressure. The specific surface area of TE-G is 1055.7 m^2^ g^−1^, and a big specific surface area facilitates the infiltration and stripping of TE-G. When increased the amount of KMnO_4_, abundant MnO_2_ nanosheets loading on graphene surface decreases the specific surface area. The specific surfaces of MnO_2_/G-5, MnO_2_/G-10, and MnO_2_/G-20 are 252.3 m^2^ g^−1^, 76.1 m^2^ g^−1,^ and 39.4 m^2^ g^−1^. From Figure 6B, TE-G has much more micropores and mesopores. The loading of MnO_2_ on graphene decreases the fraction of micropores and mesopores. However, the macroporous structure of MnO_2_/G composite would accelerate the charge transfer and ion diffusion, further improving the electrochemical performance.

### Electrochemical Performance

**FIGURE 7:**
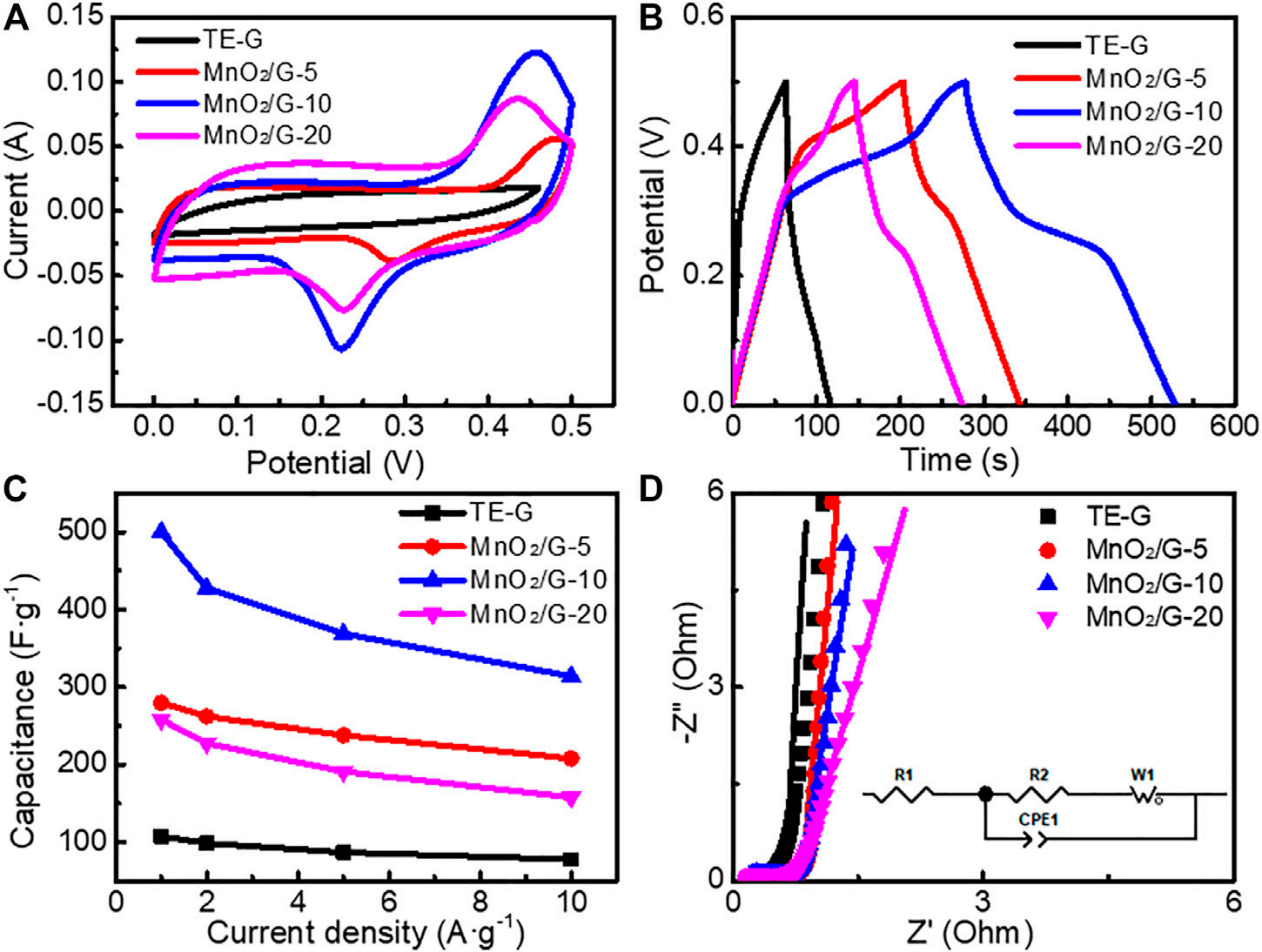
Electrochemical performance of TE-G and different MnO_2_/G composites in a three-electrode cell **(A)** CV curves tested at 20 mV s−1 **(B)** the charge-discharge curves tested at 1 A g^−1^ **(C)** the specific capacitance **(D)** EIS plots, equivalent circuit and fitting curves (solid line).

A three-electrode system was used to investigate the electrochemical performance of different samples, by using 6 M KOH electrolyte. Figure 7A presents the CV curves of TE-G and different MnO_2_/G composites at 20 mV s^−1^. The CV curve of TE-G displays a quasi-rectangular shape, reflecting the EDLC characteristic of TE-G. When introducing MnO_2_, the resulting MnO_2_/G composites show two pseudocapacitive peaks of MnO_2_ corresponding to the faradic redox reaction of MnO_2_. The faradic redox reaction mechanism of MnO_2_ is verified as the valence shift between Mn^4+^/Mn^3+^ and Mn^3+^/Mn^2+^ (Zhou et al., 2015; Xie et al., 2019). The redox peaks centered at ∼0.1 and ∼0.4 V (vs Hg/HgO) can be assigned to the reversible redox reaction: Mn^4+^ ↔ Mn^3+^ + e^−^, while the other pair of redox peaks around ∼0.3 and ∼0.6 V (vs Hg/HgO) originate from the faradic redox reactions related to Mn^3+^↔ Mn^2+^ + e^−^ (Toupin et al., 2004; Zhou et al., 2015; Xie et al., 2019), corresponding to the two faradic redox peaks in CV curve further reflecting the pseudocapacitance characteristics from MnO_2._ In addition, the CV curve of the MnO_2_/G-10 sample has the largest area among these samples, revealing the maximum specific capacitance. Figure 7B exhibits the GCD curves of TE-G and different MnO_2_/G samples. TE-G shows a linear symmetrical triangle, reflecting a typical EDLCs feature related to the adsorption and desorption of ions. When increased the amount of KMnO_4_, the pseudocapacitive feature can be verified by the shape of GCD curves. The MnO_2_/G-10 composite exhibits the longest discharge time of 250.0 s, much longer than that of pure TE-G (53.8s). On the basis of the equation of SC = I·Δt/(mV), the specific capacitance would be obtained. From Figure 7C, TE-G has a specific capacitance of 107.6 F g^−1^ at 1 A g^−1^. When hybridizing with MnO_2_, MnO_2_/G composites show high specific capacitances. Among these composites, the MnO_2_/G-10 sample has the maximum specific capacitance of 500 F g^−1^ at 1 A g^−1^. Even operated at 10 A g^−1^, the capacitance is 314 F g^−1^, which is assigned to a large number of MnO_2_ nanosheets with high capacitance content loaded to the surface of graphene. In comparison with MnO_2_/G-10, the MnO_2_/G-20 composite has a capacitance of 158 F g^−1^, because of the stacked MnO_2_ aggregation on graphene (Figure 2E). The MnO_2_ aggregations obstruct the fast transfer of charges/ions, further decreasing the capacitance. Therefore, the MnO_2_/G-20 composite exhibit a lower specific capacitance and poor electrochemical performance.

Figure.7D shows the EIS plots of different samples. Each EIS curve consists of an oblique line in the low-frequency range and a hemisphere in the high-frequency range. The Warburg impedance (W1) can be reflected by an oblique line, which reflects the diffusive resistance of the electrode in the electrolyte. The intercept in the *X*-axis and the diameter of the hemisphere reflect the internal resistance (R1) and charge transfer resistance (R2), respectively. ZView software was used to obtain the fitting curves (solid line) in Figure.7D. The fitting data were listed in Supplementary Table S1. The R1 values of TE-G, MnO_2_/G-5, MnO_2_/G-10, and MnO_2_/G-20 are 0.484, 0.480, 0.213, and 0.217 Ω, respectively. In addition, the R2 values are 0.341, 0.669, 0.332 and 0.379 Ω, respectively. Therefore, the MnO_2_/G-10 composite exhibits the minimum value of R1 and R2 among these samples, which indicates the minimum internal resistance and charge transfer resistance. The reason can be explained as the residual graphene in composite enhances the electronic conductivity. Moreover, hierarchical porous flower spheres of MnO_2_ promote the fast transfer of charges/ions, which facilitate the pseudocapacitive reaction of MnO_2_ in the electrolyte. Unfortunately, abundant MnO_2_ aggregated clusters impede the rapid transfer of charges/ions, increase the internal resistance, which leads to the poor electrochemical performance of KMnO_4_/G-20 composite.

To further verify the performance advantage of MnO_2_/G composite, the electrochemical performance of MnO_2_/rGO composites are given in Supplementary Figure S4. Both CV curves and GCD curves of different MnO_2_/rGO composites show the pseudocapacitive feature of MnO_2,_ the area of MnO_2_/rGO composites enclosed by the CV curve is much smaller than that of the MnO_2_/G-10 composite. In addition, the maximum discharge time of MnO_2_/rGO-20 is 64.7 s, the specific capacitance can be calculated as 129.4 F g^−1^, much lower than that of the MnO_2_/G composite. The reason is the less carbon fraction in rGO limits the redox reaction with KMnO_4_, resulting in less MnO_2_ nanosheets loading on rGO (in Supplementary Figure S2). Therefore, TE-G shows an obvious performance advantage to high-cost rGO.

**FIGURE 8:**
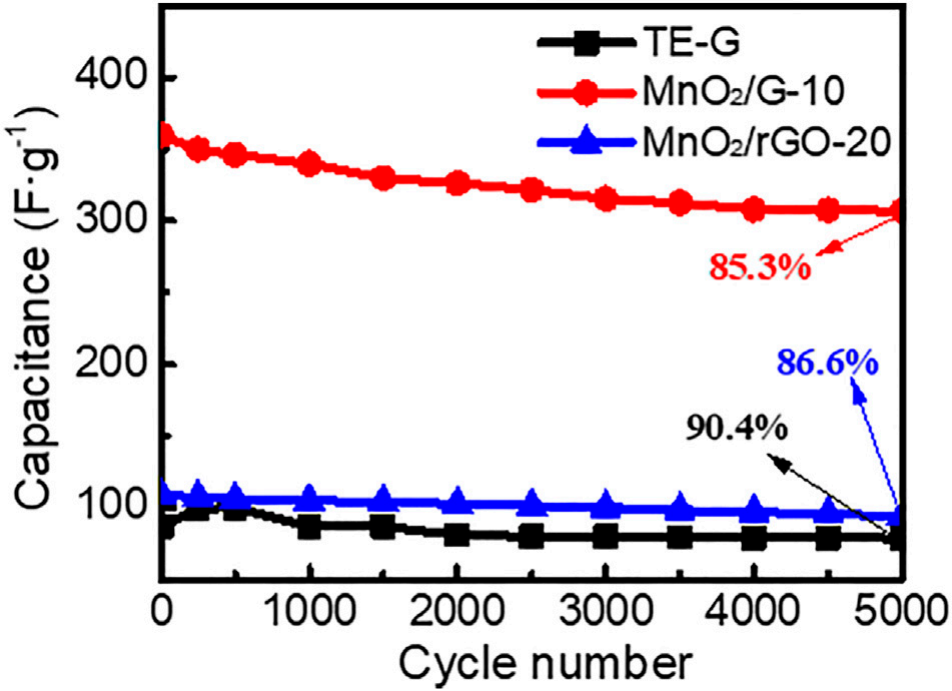
Cycling stability of TE-G, MnO_2_/G-10and MnO_2_/rGO-20 composites electrode tested in three-electrode system.

The cycling stability of TE-G, MnO_2_/G-10, and MnO_2_/rGO-20 composite was tested at a current density of 5 A g^−1^. As given in Figure 8, the specific capacitance of TE-G increases and then decreases during the first 500 cycles, which is assigned to poor wettability between TE-G and the electrolyte. As TE-G only contains a small amount of oxygen-containing groups, the wettability between TE-G and the electrolyte is poor. With the progress of the charge-discharge cycle, the wettability between TE-G and the electrolyte is improved, and the specific capacitance gradually increases. However, due to the limitation of the material itself, the specific capacitance content of TE-G decreases gradually with the increase of the cycle numbers. The specific capacitance of TE-G declines from the original 87.0 F g^−1^ to 78.6 F g^−1^ after 5,000 cycles. The capacitance retention rate is 90.4%, for the EDLC feature. The capacitance of MnO_2_/rGO composites decreases from an initial 108.6 F g^−1^–94.1 F g^−1^, and the capacitance retention rate is 86.6%, which was attributed to the lower content of MnO_2_ and more graphene lamellar residues (as shown in Supplementary Figure S2). In comparison with MnO_2_/rGO, the MnO_2_/G-10 composite has a low capacitance retention rate of 85.3%, and the capacitance decreases to 307.0 F g^−1^ from 340.1 F g^−1^. The low capacitance retention of the MnO_2_/G-10 sample is attributed to a high fraction of MnO_2_ nanosheets in the composite. However, considering the high specific capacitance, MnO_2_/G-10 composite still presents outstanding cycling stability, which is attributed to the residual graphene layer remaining in the flower spherical structure.

To testify the outstanding performance of MnO_2_/G composite with a flower spherical structure, we list the capacitance and long-term cycling performance of reported MnO_2_/graphene in Table 1. Considering the difference in testing conditions, the MnO_2_/C electrode material has the largest specific capacitance of 480.3 F g^−1^ (Jeong et al., 2021). The maximum capacitance retention is 99.4% for 5,000 cycles (Vimuna et al., 2020). Although our MnO_2_/G composite (MnO_2_/G-10) has a low capacitance retention rate of 85.3%. Particularly, the specific capacitance of our MnO_2_/G composite is 500 F g^−1^, much higher than reported samples. Therefore, the MnO_2_/G composite with flower spheres structure displays an excellent electrochemical performance, which can be ascribed to two aspects. First of all, a large amount of MnO_2_ nanosheets arrays loading on graphene constructs a homogeneous hierarchical porous structure, which promotes the transport of electrons and ions, and reduces the charge transfer resistance. Moreover, the special microstructure facilitates the interface contact between MnO_2_ nanosheets and electrolyte and releases a high specific capacitance. Secondly, TE-G is composed of stacking graphene layers, which facilitates the redox reaction between KMnO_4_ and C, resulting in a high fraction of MnO_2_ in composite, which increases the pseudocapacitance. Moreover, the residual graphene layer in composite improves the conductivity of electrode material and decreases the internal resistance, which enables an outstanding rate capability and cycling performance.

**TABLE 1:** Summary of the electrochemical performance of existing C/MnO2 composites.

Electrode materials	Electrolyte	Capacitance (F·g^−1^)	Cycling stability	Ref
MnO_2_/RGO	1 M Na_2_SO_4_	467 at 1 A g^−1^	93.1%-2500 cycle	Zhang et al. (2020b)
MnO_2_/rGO (NMG)	1M Na_2_SO_4_	140.3 at 1 mA	99.4%-5,000 cycle	Vimuna et al. (2020)
MnO_2_@PCN	1 M Na_2_SO_4_	225 at 0.5 A g^−1^	—	Yang et al. (2020)
MnO_2_/C	1M Na_2_SO_4_	480.3 at 0.5 mAcm^−2^	71%-10000 cycle	Jeong et al. (2021)
PWC/MnO_2_/GQDs	1 M Na_2_SO_4_	188.4 at 1 mA cm^−2^	95.3%-2000 cycle	Zhang et al. (2020d)
RGO-S/MnO_2_	2.5 MKNO_3_	180.4 at 1 A g^-1^	—	Tarimo et al. (2020)
MnO_2_/GH	1 M KOH	445.7 at 0.5 A g^−1^	82.4%-5000cycle	Zhang et al. (2016)
MnO_2_/PC-Cs/MnO_2_	1 M KOH	397 at 1 A g^−1^	93.1%-5,000 cycles	Hong et al. (2021)
rGO/C/MnO_2_	3 M KOH	215.2 at 0.15 A g−1	72%-2500 cycles	Zhang et al. (2020a)
MnO_2_/G	6 M KOH	500 at 1 A g^−1^	85.3%-5,000 cycles	This work

**FIGURE 9:**
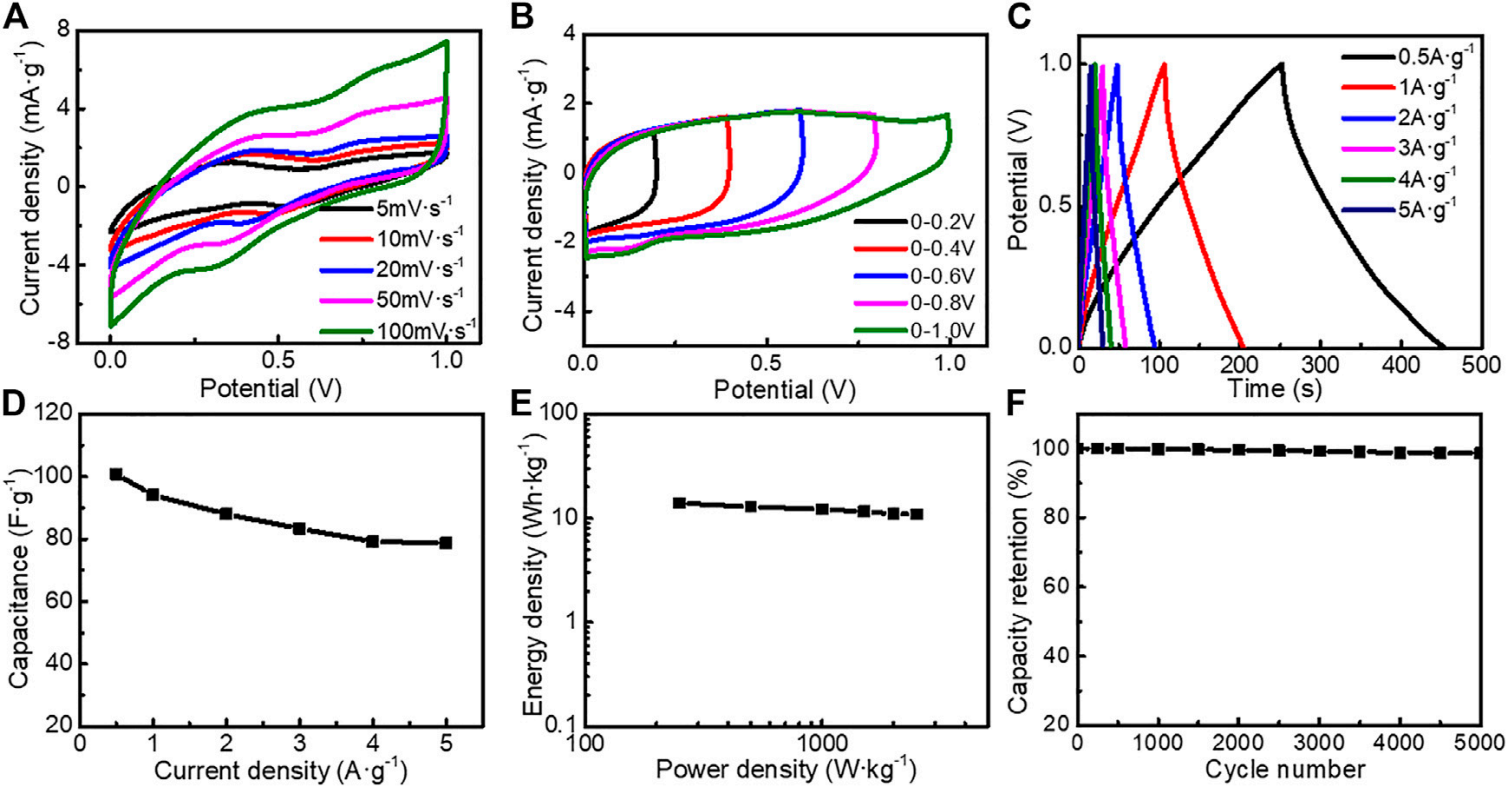
Electrochemical performance of the asymmetric supercapacitor tested in 6 M KOH electrolyte **(A)** CV curves at different scan rates **(B)** CV curves at different potential ranges **(C)** GCD curves at different current densities **(D)** GCD curves at different potential ranges **(E)** Ragone plots of the asymmetric supercapacitor **(F)** cycling stability tested at 5 A g^−1^.

In addition, we assembled an asymmetric supercapacitor (ASC) with TE-G negative electrode and MnO_2_/G-10 positive electrode. From Figure 9A, the CV curves show quasi-rectangular shapes. With an increase of scan rate, the area of the CV curve increases, with a shape of quasi-rectangular, further indicating the EDLC feature. The ASC can be operated stably under a broad voltage window of 0–1.0 V (Figure 9B). From Figure 9C, the longest discharge time reaches 201.6 s, corresponding to the maximum specific capacitance of 100.8 F g^−1^ at 0.5 A g^−1^. The capacitance reduces to 78.8 F g^−1^ at a large current density of 5 A g^−1^, indicating an excellent rate capability. Figure 9E provides the energy density (E) and power density (P) at different current densities. The energy density is 14.0 Wh kg^−1^ at the power density of 250.0 W kg^−1^. With an increase of power density, the energy density drops to 10.94 Wh kg^−1^ (2500 W kg^−1^), further reflecting the excellent power/energy combination. Figure 9F shows the cycling stability of ASC. When measured at 5 A g^−1^, the capacitance retention rate is 98.4% after 5,000 cycles, reflecting the superior cycling stability. Therefore, flower-like MnO_2_/G microspheres exhibit outstanding performance in ASC.

## Conclusion

To hybridize thermally-exfoliated graphite (TE-G) and MnO_2_, a KMnO_4_-assisted hydrothermal method was adopted to achieve the exfoliation of TE-G and the loading of MnO_2_ nanosheets. Through changing the ratio of TE-G and KMnO_4_, flower-like MnO_2_/G microspheres (MnO_2_/G-10) were fabricated containing 83.2% MnO_2_ and 16.8% residual graphene layer. To confirm the advantage of TE-G reactants, corresponding MnO_2_/rGO composites were prepared by using rGO as raw materials. When tested in a three-electrode system, the MnO_2_/G-10 sample displays a maximum specific capacitance of 500 F g^−1^, an outstanding rate of performance, and a high capacitance retention rate (85.3% for 5,000 cycles). The performance is much better than that of the optimized MnO_2_/rGO composite. The reason can be explained as the high carbon fraction in TE-G resulting in a high fraction of MnO_2_ in flower-like MnO_2_/G microspheres, and the oxygen-containing groups in rGO reduce the effective redox reaction between KMnO_4_ and carbon. The superior electrochemical performance of MnO_2_/G-10 is related to the hierarchical porous structure constructed by MnO_2_ nanosheet arrays and conductive graphene in the composite. Moreover, the ASC consisted of MnO_2_/G positive electrode and TE-G negative electrode has a capacitance of 100.8 F g^−1^ at 0.5A g^−1^, with a high capacitance retention of 98.6% for 5,000 cycles. The energy density is 14.0 Wh kg^−1^ at the power density of 250.0 W kg^−1^. In consideration of the low cost of raw materials, the MnO_2_/G composite shows great application potential in the supercapacitors field.

## Data Availability

The original contributions presented in the study are included in the article/Supplementary Material, further inquiries can be directed to the corresponding author.

## References

[B1] Brousse T. Taberna P.-L. Crosnier O. Dugas R. Guillemet P. Scudeller Y. (2007). Long-term Cycling Behavior of Asymmetric Activated carbon/MnO2 Aqueous Electrochemical Supercapacitor. J. Power Sourc. 173, 633–641. 10.1016/j.jpowsour.2007.04.074

[B2] Ferrero G. A. Sevilla M. Fuertes A. B. (2015). Mesoporous Carbons Synthesized by Direct Carbonization of Citrate Salts for Use as High-Performance Capacitors. Carbon 88, 239–251. 10.1016/j.carbon.2015.03.014

[B3] Gong D. Tong H. Xiao J. Li T. Liu J. Wu Y. (2021). Self-standing Manganese Dioxide/graphene Carbon Nanotubes Film Electrode for Symmetric Supercapacitor with High Energy Density and superior Long Cycling Stability. Ceramics Int. 47, 33020–33027. 10.1016/j.ceramint.2021.08.202

[B4] Hong X. Wang X. Li Y. Fu J. Liang B. (2021). Sandwich Structured MnO2/carbon nanosheet/MnO2 Composite for High-Performance Supercapacitors. J. Alloys Compd. 889, 161821. 10.1016/j.jallcom.2021.161821

[B5] Jeong J. M. Park S. H. Park H. J. Jin S. B. Son S. G. Moon J. M. (2021). Alternative‐Ultrathin Assembling of Exfoliated Manganese Dioxide and Nitrogen‐Doped Carbon Layers for High‐Mass‐Loading Supercapacitors with Outstanding Capacitance and Impressive Rate Capability. Adv. Funct. Mater. 31, 2009632. 10.1002/adfm.202009632

[B6] Lei R. Gao J. Qi L. Ye L. Wang C. Le Y. (2020). Construction of MnO2 Nanosheets@graphenated Carbon Nanotube Networks Core-Shell Heterostructure on 316L Stainless Steel as Binder-free Supercapacitor Electrodes. Int. J. Hydrogen Energ. 45, 28930–28939. 10.1016/j.ijhydene.2019.09.070

[B7] Li D. Lin J. Lu Y. Huang Y. He X. Yu C. (2020). MnO2 Nanosheets Grown on N-Doped Agaric-Derived Three-Dimensional Porous Carbon for Asymmetric Supercapacitors. J. Alloys Compd. 815, 152344. 10.1016/j.jallcom.2019.152344

[B8] Li K. Hu Z. Zhao R. Zhou J. Jing C. Sun Q. (2021). A Multidimensional Rational Design of Nickel-Iron Sulfide and Carbon Nanotubes on Diatomite via Synergistic Modulation Strategy for Supercapacitors. J. Colloid Interf. Sci. 603, 799–809. 10.1016/j.jcis.2021.06.131 34246089

[B9] Liu X. Liang B. Long J. (2021). Preparation of Novel Thick Sheet Graphene and its Effect on the Properties of Polyolefins with Different Crystallinities. Polym. Bull. 10.1007/s00289-021-03791-x

[B10] Liu Y. He D. Wu H. Duan J. Zhang Y. (2015). Hydrothermal Self-Assembly of Manganese Dioxide/Manganese Carbonate/Reduced Graphene Oxide Aerogel for Asymmetric Supercapacitors. Electrochimica Acta 164, 154–162. 10.1016/j.electacta.2015.01.223

[B11] Liu Y. Yan D. Li Y. Wu Z. Zhuo R. Li S. (2014). Manganese Dioxide Nanosheet Arrays Grown on Graphene Oxide as an Advanced Electrode Material for Supercapacitors. Electrochimica Acta 117, 528–533. 10.1016/j.electacta.2013.11.121

[B12] Oncu A. Cetinkaya T. Akbulut H. (2021). Enhancement of the Electrochemical Performance of Free-Standing Graphene Electrodes with Manganese Dioxide and Ruthenium Nanocatalysts for Lithium-Oxygen Batteries. Int. J. Hydrogen Energ. 46, 17173–17186. 10.1016/j.ijhydene.2021.02.154

[B13] Ping Y. Liu Z. Li J. Han J. Yang Y. Xiong B. (2019). Boosting the Performance of Supercapacitors Based Hierarchically Porous Carbon from Natural Juncus Effuses by Incorporation of MnO2. J. Alloys Compd. 805, 822–830. 10.1016/j.jallcom.2019.07.125

[B14] Raj C. J. Manikandan R. Cho W.-J. Yu K. H. Kim B. C. (2020). High-performance Flexible and Wearable Planar Supercapacitor of Manganese Dioxide Nanoflowers on Carbon Fiber Cloth. Ceramics Int. 46, 21736–21743. 10.1016/j.ceramint.2020.05.282

[B15] Sevilla M. Fuertes A. B. (2014). Direct Synthesis of Highly Porous Interconnected Carbon Nanosheets and Their Application as High-Performance Supercapacitors. ACS Nano 8, 5069–5078. 10.1021/nn501124h 24731137

[B16] Sha Z. Huang F. Zhou Y. Zhang J. Wu S. Chen J. (2021). Synergies of Vertical Graphene and Manganese Dioxide in Enhancing the Energy Density of Carbon Fibre-Based Structural Supercapacitors. Composites Sci. Technol. 201, 108568. 10.1016/j.compscitech.2020.108568

[B17] Srimuk P. Luanwuthi S. Krittayavathananon A. Sawangphruk M. (2015). Solid-type Supercapacitor of Reduced Graphene Oxide-Metal Organic Framework Composite Coated on Carbon Fiber Paper. Electrochimica Acta 157, 69–77. 10.1016/j.electacta.2015.01.082

[B18] Tarimo D. J. Oyedotun K. O. Mirghni A. A. Sylla N. F. Manyala N. (2020). High Energy and Excellent Stability Asymmetric Supercapacitor Derived from sulphur-reduced Graphene Oxide/manganese Dioxide Composite and Activated Carbon from Peanut Shell. Electrochimica Acta 353, 136498. 10.1016/j.electacta.2020.136498

[B19] Thommes M. Cychosz K. A. (2014). Physical Adsorption Characterization of Nanoporous Materials: Progress and Challenges. Adsorption 20, 233–250. 10.1007/s10450-014-9606-z

[B20] Toupin M. Brousse T. Bélanger D. (2004). Charge Storage Mechanism of MnO2 Electrode Used in Aqueous Electrochemical Capacitor. Chem. Mater. 16, 3184–3190. 10.1021/cm049649j

[B21] Vimuna V. M. Athira A. R. Dinesh Babu K. V. Xavier T. S. (2020). Simultaneous Stirring and Microwave Assisted Synthesis of Nanoflakes MnO2/rGO Composite Electrode Material for Symmetric Supercapacitor with Enhanced Electrochemical Performance. Diamond Relat. Mater. 110, 108129. 10.1016/j.diamond.2020.108129

[B22] Wang J. Yang H. Sun Q. Zhou C. Zhang X. Ge L. (2021a). Synthesis of δ-MnO2/C Assisted with Carbon Sheets by Directly Carbonizing from Corn Stalk for High-Performance Supercapacitor. Mater. Lett. 285, 129116. 10.1016/j.matlet.2020.129116

[B23] Wang T. Li K. Le Q. Zhu S. Guo X. Jiang D. (2021b). Tuning Parallel Manganese Dioxide to Hollow Parallel Hydroxyl Oxidize Iron Replicas for High-Performance Asymmetric Supercapacitors. J. Colloid Interf. Sci. 594, 812–823. 10.1016/j.jcis.2021.03.075 33794403

[B24] Wang X. Chen S. Li D. Sun S. Peng Z. Komarneni S. (2018). Direct Interfacial Growth of MnO2 Nanostructure on Hierarchically Porous Carbon for High-Performance Asymmetric Supercapacitors. ACS Sustain. Chem. Eng. 6, 633–641. 10.1021/acssuschemeng.7b02960

[B25] Wei C. Xu C. Li B. Du H. Kang F. (2012). Preparation and Characterization of Manganese Dioxides with Nano-Sized Tunnel Structures for Zinc Ion Storage. J. Phys. Chem. Sol. 73, 1487–1491. 10.1016/j.jpcs.2011.11.038

[B26] Xie Y. Yang C. Chen P. Yuan D. Guo K. (2019). MnO2-decorated Hierarchical Porous Carbon Composites for High-Performance Asymmetric Supercapacitors. J. Power Sourc. 425, 1–9. 10.1016/j.jpowsour.2019.03.122

[B27] Xu M.-W. Zhao D.-D. Bao S.-J. Li H.-L. (2007). Mesoporous Amorphous MnO2 as Electrode Material for Supercapacitor. J. Solid State. Electrochem. 11, 1101–1107. 10.1007/s10008-006-0246-4

[B28] Xu Z. Sun S. Cui W. Lv J. Geng Y. Li H. (2018). Interconnected Network of Ultrafine MnO2 Nanowires on Carbon Cloth with weed-like Morphology for High-Performance Supercapacitor Electrodes. Electrochimica Acta 268, 340–346. 10.1016/j.electacta.2018.02.138

[B29] Yan C. Tong X. Qu Y. Zhou Y. Pang N. Xu S. (2021). Porous Manganese Dioxide Nanosheets on Modified Graphite Felt for Cathodes in High-Capacity Flexible Zinc-MnO2 Batteries. Vacuum 191, 110353. 10.1016/j.vacuum.2021.110353

[B30] Yan D. Zhang H. Li S. Zhu G. Wang Z. Xu H. (2014). Formation of Ultrafine Three-Dimensional Hierarchical Birnessite-type MnO2 Nanoflowers for Supercapacitor. J. Alloys Compd. 607, 245–250. 10.1016/j.jallcom.2014.04.077

[B31] Yang G. Park S.-J. (2018). MnO2 and Biomass-Derived 3D Porous Carbon Composites Electrodes for High Performance Supercapacitor Applications. J. Alloys Compd. 741, 360–367. 10.1016/j.jallcom.2018.01.108

[B32] Yang W. Gao Z. Wang J. Wang B. Liu Q. Li Z. (2012). Synthesis of Reduced Graphene Nanosheet/urchin-like Manganese Dioxide Composite and High Performance as Supercapacitor Electrode. Electrochimica Acta 69, 112–119. 10.1016/j.electacta.2012.02.081

[B33] Yang Y. Niu H. Qin F. Guo Z. Wang J. Ni G. (2020). MnO2 Doped Carbon Nanosheets Prepared from Coal Tar Pitch for Advanced Asymmetric Supercapacitor. Electrochimica Acta 354, 136667. 10.1016/j.electacta.2020.136667

[B34] Zhang H. Lin L. Wu B. Hu N. (2020a). Vertical Carbon Skeleton Introduced Three-Dimensional MnO2 Nanostructured Composite Electrodes for High-Performance Asymmetric Supercapacitors. J. Power Sourc. 476, 228527. 10.1016/j.jpowsour.2020.228527

[B35] Zhang M. Yang D. Li J. (2020b). Effective Improvement of Electrochemical Performance of Electrodeposited MnO2 and MnO2/reduced Graphene Oxide Supercapacitor Materials by Alcohol Pretreatment. J. Energ. Storage 30, 101511. 10.1016/j.est.2020.101511

[B36] Zhang M. Zheng H. Zhu H. Xu Z. Liu R. Chen J. (2020c). Graphene-wrapped MnO2 Achieved by Ultrasonic-Assisted Synthesis Applicable for Hybrid High-Energy Supercapacitors. Vacuum 176, 109315. 10.1016/j.vacuum.2020.109315

[B37] Zhang N. Fu C. Liu D. Li Y. Zhou H. Kuang Y. (2016). Three-Dimensional Pompon-like MnO2/Graphene Hydrogel Composite for Supercapacitor. Electrochimica Acta 210, 804–811. 10.1016/j.electacta.2016.06.004

[B38] Zhang W. Yang Y. Xia R. Li Y. Zhao J. Lin L. (2020d). Graphene-quantum-dots-induced MnO2 with Needle-like Nanostructure Grown on Carbonized wood as Advanced Electrode for Supercapacitors. Carbon 162, 114–123. 10.1016/j.carbon.2020.02.039

[B39] Zhang W. Yuan X. Yan X. You M. Jiang H. Miao J. (2021). Tripotassium Citrate Monohydrate Derived Carbon Nanosheets as a Competent Assistant to Manganese Dioxide with Remarkable Performance in the Supercapacitor. Front. Chem. Sci. Eng. 10.1007/s11705-021-2065-7

[B40] Zhao N. Deng L. Luo D. Zhang P. (2020). One-step Fabrication of Biomass-Derived Hierarchically Porous carbon/MnO Nanosheets Composites for Symmetric Hybrid Supercapacitor. Appl. Surf. Sci. 526, 146696. 10.1016/j.apsusc.2020.146696

[B41] Zhou D. Lin H. Zhang F. Niu H. Cui L. Wang Q. (2015). Freestanding MnO2 Nanoflakes/porous Carbon Nanofibers for High-Performance Flexible Supercapacitor Electrodes. Electrochimica Acta 161, 427–435. 10.1016/j.electacta.2015.02.085

[B42] Zhu L. Wang J. Rong S. Wang H. Zhang P. (2017). Cerium Modified Birnessite-type MnO2 for Gaseous Formaldehyde Oxidation at Low Temperature. Appl. Catal. B: Environ. 211, 212–221. 10.1016/j.apcatb.2017.04.025

