# Supplementary material for: Electrochemical Performance of MnO2/Graphene Flower-like Microspheres Prepared by Thermally-Exfoliated Graphite
Source: Front Chem. 2022 Apr 8;10:870541. doi: 10.3389/fchem.2022.870541 (PMC9024236; doi:10.3389/fchem.2022.870541)
Supplement: Supplementary file 1 [file DataSheet1.docx]

Supplementary materials

Figure.S1 FTIR spectra of TE-G, GO and rGO

Figure.S2 thermogravimetric curves of MnO_2_/rGO-5, MnO_2_/rGO-20 and MnO_2_/G-10

Figure.S3 The XRD patterns of TE-G-H_2_O (black line) and TE-G-KCl (red line)


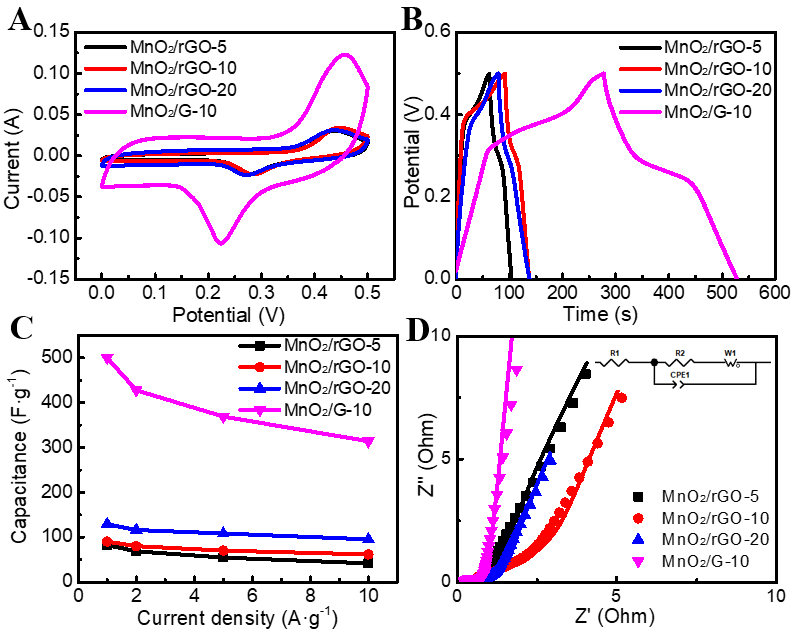


Figure.S4. Electrochemical performance of different MnO_2_/rGO composites and MnO_2_/G-10 in a three-electrode cell, (A) CV curves tested at 20 mV s^−1^; (B) the charge-discharge curves tested at 1 A g^−1^; (C) the specific capacitance, (D) EIS plots, equivalent circuit and corresponding fitting curves (solid line).

**Table S1.** Corresponding fitting results derived from Fig. 7d.

| Electrodes | R1 | R2 | W1 | CPE1 |
| --- | --- | --- | --- | --- |
| TE-G | 0.484 | 0.341 | 0.014 | 0.0003 |
| MnO_2_/G-5 | 0.480 | 0.669 | 0.573 | 0.053 |
| MnO_2_/G-10 | 0.213 | 0.332 | 1.028 | 0.002 |
| MnO_2_/G-20 | 0.217 | 0.379 | 1.547 | 0.001 |
